# Supplementary material for: Optimization of Neferine Purification Based on Response Surface Methodology and Its Anti-Metastasis Mechanism on HepG2 Cells
Source: Molecules. 2023 Jun 29;28(13):5086. doi: 10.3390/molecules28135086 (PMC10343747; doi:10.3390/molecules28135086)
Supplement: Supplementary file 1 [file molecules-28-05086-s001.zip › molecules-2429726-supplementary.pdf]

molecules

Supplementary Materials

Optimization of Neferine Purification Based on Response Surface Methodology and Its Anti-Metastasis Mechanism on HepG2 Cells

Xinzhu Wang [1], Zhenhuan Wei [1], Po Hu [1], Weibo Xia [1], Zhixin Liao [2], Israa Assani [2], Guangming Yang [1,*] and Yang Pan [1,*]

[1] School of Pharmacy, Nanjing University of Chinese Medicine, 138 Xianlin Avenue, Qixia District, Nanjing 210023, China; xzwang@njucm.edu.cn (X.W.); 20200860@njucm.edu.cn (Z.W.); hupo_cpu@foxmail.com (P.H.); xwb15366067163@163.com (W.X.)

[2] Department of Pharmaceutical Engineering, School of Chemistry and Chemical Engineering, Southeast University, Nanjing 211189, China; zxliao@seu.edu.cn (Z.L.); israaassani@hotmail.com (I.A.)

* Correspondence: ygm@njucm.edu.cn (G.Y.); ypan@njucm.edu.cn (Y.P.); Tel.: +86-137-7065-2078 (G.Y.); +86-136-0519-2035 (Y.P.)

Contents

Table S1 Column material ratio 1:20 3
Table S2 Column material ratio 1:20 34
Table S3 Column material ratio 1:20 4
Table S4 column material ratio 1:40 4
Table S5 column material ratio 1:40 5
Table S6 column material ratio 1:40 5
Table S7 column material ratio 1:60 5
Table S8 column material ratio 1:60 6
Table S9 column material ratio 1:60 6
Table S10 column material ratio 1:80 6
Table S11 column material ratio 1:80 7
Table S12 column material ratio 1:80 7
Table S13 flow rate 1ml/min 7
Table S14 flow rate 1ml/min 8
Table S15 flow rate 1ml/min 8
Table S16 flow rate 2ml/min 8
Table S17 flow rate 2ml/min 9
Table S18 flow rate 2ml/min 9
Table S19 flow rate 3ml/min 9
Table S20 flow rate 3ml/min 10
Table S21 flow rate 3ml/min 10
Figure S1 the 1H NMR (500 MHz, MEOD) spectrum of neferine 11
Figure S2 Mass spectrometric data of neferine 11
Table S22 inhibition of HepG2 proliferation activity by 96 h neferine 11
Table S23 inhibition of HepG2 proliferation activity by 72 h neferine 12
Table S24 inhibition of HepG2 proliferation activity by 48 h neferine 12
Table S25 inhibition of HepG2 proliferation activity by 24 h neferine 12
Table S26 cell scratch assay 13
Table S27 transwell migration rate 13
Table S28 transwell invasion rate 13
Figure S3 p-MYPT1 14
Figure S4 RhoA 15
Figure S5 Vimentin 16
Figure S6 β-Actin 17
Table S29 Grayscale analysis of RhoA 18
Table S30 Grayscale analysis of Vimentin 18
Table S31 Hydrogen spectrum data of neferine 19

Table S1 Column material ratio 1:20

Purity at 282 nm	Elution gradient	Peak area (mAU * s)	Yield (mg)	Yield (%)
0	100% dichloromethane	0.00		
0	70%dichloromethane&30%methanol	0.00		
0		0.00		
0		0.00		
0	60%dichloromethane&40%methanol	0.00		
0		0.00		
0		0.00		
0		0.00		
0		0.00		
0		0.00		
71.9		9587.90	1.1363	0.57
89.18		52329.10	5.9785	2.99
78.13		49196.20	5.6235	2.81
30.56		26549.20	3.0578	1.53
3.04		427.47	0.0985	0.05
1.04		55.60	0.0563	0.03
0.95		34.16	0.0539	0.03
0	50%dichloromethane&50%methanol	0.00		
0		0.00		
0		0.00		
0	100%dichloromethane	0.00		

Table S2 Column material ratio 1:20

Purity at 282 nm	Elution gradient	Peak area (mAU * s)	Yield (mg)	Yield (%)
0	100%dichloromethane	0.00		
0	70%dichloromethane&30%methanol	0.00		
0	60%dichloromethane&40%methanol	0.00		
0		0.00		
0		0.00		
0		0.00		
73.2602		55397.80	6.3261	3.16
44.5307		37800.30	4.3325	2.17
10.054		2919.02	0.3807	0.19
5.4845		1882.84	0.2634	0.13
2.2199		1625.94	0.2342	0.12
0.14		42.18	0.0548	0.03
0		0.00		
0	50%dichloromethane&50%methanol	0.00		
0	100%dichloromethane	0.00		

Table S3 Column material ratio 1:20

Purity at 282 nm	Elution gradient	Peak area (mAU * s)	Yield (mg)	Yield (%)
0	100%dichloromethane	0.00		
0.5011	70%dichloromethane&30%methanol	10.46	0.0512	0.03
0	70%dichloromethane&30%methanol	0.00		
0	60%dichloromethane&40%methanol	0.00		
0		0.00		
9.3558		84.72	0.0596	0.03
79.4103		44629.60	5.1062	2.55
80.6187		34315.70	3.9377	1.97
75.9435		74449.30	8.4845	4.24
33.1532		25477.20	2.9364	1.47
3.6162		447.54	0.1007	0.05
5.8585	50%dichloromethane&50%methanol	21.76	0.0525	0.03

Table S4 column material ratio 1:40

Purity at 282 nm	Elution gradient	Peak area (mAU * s)	Yield (mg)	Yield (%)
0	100%dichloromethane	0.00		
27.17	70%dichloromethane&30%methanol	123.37	0.0640	0.06
0		0.00		
0	70%dichloromethane&30%methanol	0.00		
0	60%dichloromethane&40%methanol	0.00		
1.27	60%dichloromethane&40%methanol	13.43	0.0516	0.05
0		0.00		
78.26		14258.20	1.6654	1.67
70.71		69867.80	7.9655	7.97
9.47		1433.52	0.2124	0.21
0	50%dichloromethane&50%methanol	0.00		
0		0.00		

Table S5 column material ratio 1:40

Purity at 282 nm	Elution gradient	Peak area (mAU * s)	Yield (mg)	Yield (%)
0	100%dichloromethane	0.00		
0	70%dichloromethane&30%methanol	0.00		
0	60%dichloromethane&40%methanol	0.00		
0		0.00		
0		0.00		
0		0.00		
73.099		83270.10	9.4838	9.48
66.2209		24672.80	2.8453	2.85
55.4664		33889.10	3.8894	3.89
27.1039		18299.20	2.1232	2.12
4.1805		520.36	0.1090	0.11
0	50%dichloromethane&50%methanol	0.00		

Table S6 column material ratio 1:40

Purity at 282 nm	Elution gradient	Peak area (mAU * s)	Yield (mg)	Yield (%)
0	100%dichloromethane	0.00		
0	70%dichloromethane&30%methanol	0.00		
0	60%dichloromethane&40%methanol	0.00		
54.0215		6307.00	0.7646	0.76
76.6173		14627.60	1.7072	1.71
78.8763		21532.20	2.4895	2.49
75.0346		64424.80	7.3488	7.35
26.346		8282.65	0.9884	0.99
24.9356		5191.48	0.6382	0.64
0		0.00		
0	50%dichloromethane&50%methanol	0.00		

Table S7 column material ratio 1:60

Purity at 282 nm	Elution gradient	Peak area (mAU * s)	Yield (mg)	Yield (%)
0	100%dichloromethane	0.00		
0	70%dichloromethane&30%methanol	0.00		
0	70%dichloromethane&30%methanol	0.00		
0	70%dichloromethane&30%methanol	0.00		
14.19	60%dichloromethane&40%methanol	605.31	0.1186	0.18
64.14	60%dichloromethane&40%methanol	43383.60	4.9650	7.45
28.5		5934.85	0.7224	1.08
3.06		167.19	0.0690	0.10
0	50%dichloromethane&50%methanol	0.00		
0		0.00		
0	100%dichloromethane	0.00		

Table S8 column material ratio 1:60

Purity at 282 nm	Elution gradient	Peak area (mAU * s)	Yield (mg)	Yield (%)
0	100%dichloromethane	0.00		
0	70%dichloromethane&30%methanol	0.00		
0	70%dichloromethane&30%methanol	0.00		
0	60%dichloromethane&40%methanol	0.00		
0	60%dichloromethane&40%methanol	0.00		
67.3353	60%dichloromethane&40%methanol	14224.90	1.6616	2.49
72.9551		39216.70	4.4930	6.74
39.6955		4355.84	0.5435	0.82
0	50%dichloromethane&50%methanol	0.00		

Table S9 column material ratio 1:60

Purity at 282 nm	Elution gradient	Peak area (mAU * s)	Yield (mg)	Yield (%)
0	100%dichloromethane	0.00		
0		0.00		
0	70%dichloromethane&30%methanol	0.00		
0	60%dichloromethane&40%methanol	0.00		
17.9707		726.72	0.1324	0.20
67.8686		39337.40	4.5066	6.76
65.388		31982.50	3.6734	5.51
27.7071		5670.72	0.6925	1.04
5.3417		568.69	0.1145	0.17
0	50%dichloromethane&50%methanol	0.00		

Table S10 column material ratio 1:80

Purity at 282 nm	Elution gradient	Peak area (mAU * s)	Yield (mg)	Yield (%)
0	100%dichloromethane	0.00		
0		0.00		
0	70%dichloromethane&30%methanol	0.00		
0		0.00		
0	60%dichloromethane&40%methanol	0.00		
0		0.00		
58.024		14489.60	1.6916	3.38
69.866		41525.70	4.7545	9.51
13.468		1588.54	0.2300	0.46
0		0.00		
9.226	50%dichloromethane&50%methanol	12.84	0.0515	0.10
0		0.00		

Table S11 column material ratio 1:80

Purity at 282 nm	Elution gradient	Peak area (mAU * s)	Yield (mg)	Yield (%)
0	100%dichloromethane	0.00		
0	70%dichloromethane&30%methanol	0.00		
0	60%dichloromethane&40%methanol	0.00		
43.2527		496.93	0.1063	0.21
75.2405	60%dichloromethane&40%methanol	35876.80	4.1146	8.23
69.8217		45349.90	5.1878	10.38
33.2456		9768.68	1.1567	2.31
2.1681		124.92	0.0642	0.13
0		0.00		
0	50%dichloromethane&50%methanol	0.00		
0	100%dichloromethane	0.00		

Table S12 column material ratio 1:80

Purity at 282 nm	Elution gradient	Peak area (mAU * s)	Yield (mg)	Yield (%)
0	100%dichloromethane	0.00		
0	70%dichloromethane&30%methanol	0.00		
0	60%dichloromethane&40%methanol	0.00		
42.2438		45350.70	5.1879	10.38
49.723	60%dichloromethane&40%methanol	19868.40	2.3010	4.60
19.6301		1373.72	0.2057	0.41
5.089		409.12	0.0964	0.19
5.598		116.40	0.0632	0.13
9.574	50%dichloromethane&50%methanol	12.67	0.0515	0.10

Table S13 flow rate 1ml/min

Purity at 282 nm	Elution gradient	Peak area (mAU * s)	Yield (mg)	Yield (%)
0	100%dichloromethane	0.00		
0	70%dichloromethane&30%methanol	0.00		
0	60%dichloromethane&40%methanol	0.00		
0	50%dichloromethane&50%methanol	0.00		
34.566	100%methanol	0.00		
79.327		0.00		
68.55		14489.60	1.6916	1.69
42.792		41525.70	4.7545	4.75

Table S14 flow rate 1ml/min

Purity at 282 nm	Elution gradient	Peak area (mAU * s)	Yield (mg)	Yield (%)
0	70%dichloromethane&30%methanol	0.00		
0	60%dichloromethane&40%methanol	0.00		
0	50%dichloromethane&50%methanol	0.00		
0	100%methanol	0.00		
8.9634		100.37	0.0614	0.06
11.3694		89.14	0.0601	0.06
3.2546		12.41	0.0514	0.05
0	70%dichloromethane&30%methanol	0.00		

Table S15 flow rate 1ml/min

Purity at 282 nm	Elution gradient	Peak area (mAU * s)	Yield (mg)	Yield (%)
0	100%dichloromethane	0.00		
0	70%dichloromethane&30%methanol	0.00		
0	50%dichloromethane&50%methanol	0.00		
0	100%methanol	0.00		
0	60%dichloromethane&40%methanol	0.00		
0	50%dichloromethane&50%methanol	0.00		
0	100%methanol	0.00		
7.7913	70%dichloromethane&30%methanol	82.83	0.0594	0.06

Table S16 flow rate 2ml/min

Purity at 282 nm	Elution gradient	Peak area (mAU * s)	Yield (mg)	Yield (%)
0	100%dichloromethane	0.00		
0		0.00		
0	70%dichloromethane&30%methanol	0.00		
40.7102		388.49	0.0941	0.09
1.0836		26.53	0.0530	0.05
0		0.00		
24.9193		1103.59	0.1751	0.18
75.1745		4981.20	0.6144	0.61
89.3838		12329.90	1.4469	1.45
82.1309		8153.71	0.9738	0.97
49.0774		17857.20	2.0731	2.07
73.6775	60%dichloromethane&40%methanol	1286.02	0.1957	0.20
0		0.00		
0	50%dichloromethane&50%methanol	0.00		
0		0.00		

Table S17 flow rate 2ml/min

Purity at 282 nm	Elution gradient	Peak area (mAU * s)	Yield (mg)	Yield (%)
0	100%dichloromethane	0.00		
0	70%dichloromethane&30%methanol	0.00		
6.5041	60%dichloromethane&40%methanol	274.04208	0.0811	0.08
35.7544		1077.8811	0.1722	0.17
27.7887		3170.41	0.4092	0.41
5.1587		134.30888	0.0653	0.07
0	50%dichloromethane&50%methanol	0.00		

Table S18 flow rate 2ml/min

Purity at 282 nm	Elution gradient	Peak area (mAU * s)	Yield (mg)	Yield (%)
0	100%dichloromethane	0.00		
0		0.00		
0	70%dichloromethane&30%methanol	0.00		
0	60%dichloromethane&40%methanol	0.00		
0		0.00		
14.783		17.71	0.0520	0.05
0		0.00		
79.7002		39758.00	4.5543	4.55
83.8159		18844.40	2.1850	2.18
80.2273		43248.10	4.9497	4.95
35.4726		33550.10	3.8510	3.85
7.4524	60%dichloromethane&40%methanol	1127.31	0.1778	0.18
2.1651	50%dichloromethane&50%methanol	7.30	0.0509	0.05
0	100%dichloromethane	0.00		

Table S19 flow rate 3ml/min

Purity at 282 nm	Elution gradient	Peak area (mAU * s)	Yield (mg)	Yield (%)
0	100%dichloromethane	0.00		
0		0.00		
0	70%dichloromethane&30%methanol	0.00		
25.2819		345.11	0.0891	0.09
75.0719		36476.20	4.1825	4.18
13.6902		1227.88	0.1892	0.19
0	60%dichloromethane&40%methanol	0.00		
0		0.00		
87.8452	50%dichloromethane&50%methanol	6001.13	0.7299	0.73
0		0.00		
0	100%dichloromethane	0.00		

Table S20 flow rate 3ml/min

Purity at 282 nm	Elution gradient	Peak area (mAU * s)	Yield (mg)	Yield (%)
0	100%dichloromethane	0.00		
0		0.00		
66.7004	70%dichloromethane&30%methanol	1116.02	0.1765	0.18
60.6318		3496.42	0.4462	0.45
52.6968		9007.40	1.0705	1.07
66.4719		23613.00	2.7252	2.73
64.0383		53685.60	6.1322	6.13
46.7331		25737.50	2.9659	2.97
15.7174		12401.60	1.4550	1.46
2.1474		263.66	0.0799	0.08
0.8532		47.35	0.0554	0.06
0	60%dichloromethane&40%methanol	0.00	0.0500	0.05
0	50%dichloromethane&50%methanol	0.00		

Table S21 flow rate 3ml/min

Purity at 282 nm	Elution gradient	Peak area (mAU * s)	Yield (mg)	Yield (%)
0	100%dichloromethane	0.00		
0		0.00		
0	70%dichloromethane&30%methanol	0.00		
65.7714		3681.81	0.4672	0.47
89.6059		5650.90	0.6902	0.69
82.0736		16842.10	1.9581	1.96
84.1852		44788.50	5.1242	5.12
71.697		29220.70	3.3605	3.36
33.3266		13395.60	1.5676	1.57
8.0285		1769.09	0.2505	0.25
2.656		151.12	0.0672	0.07
0	60%dichloromethane&40%methanol	0.00		
0	50%dichloromethane&50%methanol	0.00		

Figure S1 the 1H NMR (500 MHz, MEOD) spectrum of neferine

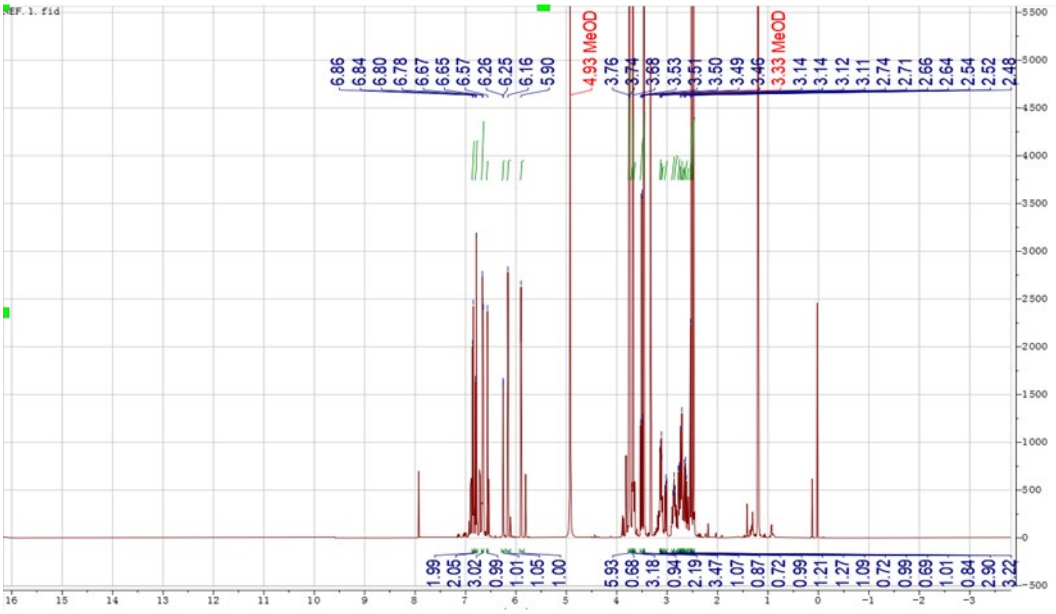

Figure S2 Mass spectrometric data of neferine

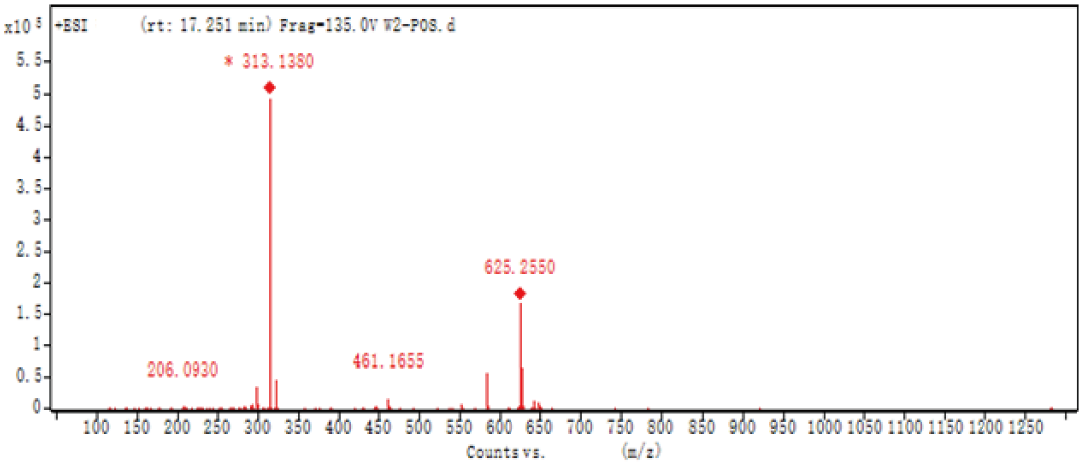

Table S22 inhibition of HepG2 proliferation activity by 96 h neferine

Neferine （μM）	96h		
0	0	0	0
10	83.9304	76.45706	72.10031
20	100.1024	85.50613	85.10972
30	100.6141	85.96626	85.03135
40	100.4094	85.65951	85.18809
50	100.1024	85.58282	85.34483

Table S23 inhibition of HepG2 proliferation activity by 72 h neferine

Neferine（μM）	72h		
0	0	0	0
10	21.02002	9.698558	18.40369
20	84.29457	70.83879	73.08707
30	97.37846	85.58322	86.14776
40	98.95138	86.63172	86.80739
50	99.88084	87.0249	86.74142

Table S24 inhibition of HepG2 proliferation activity by 48 h neferine

Neferine（μM）	48h		
0	0	0	0
10	21.54016	22.885469	22.9975
20	48.37951	47.99023	49.2153
30	77.06706	79.3282	82.91967
40	94.31098	96.90737	95.87311
50	98.7325	98.50547	98.73697

Table S25 inhibition of HepG2 proliferation activity by 24 h neferine

Neferine（μM）	24h		
0	0	0	0
10	21.12724	33.18401	5.129179
20	38.17077	42.69469	45.70669
30	64.3942	70.84769	72.03647
40	85.38643	86.83666	85.29635
50	85.75768	88.21502	87.5

Table S26 cell scratch assay

neferine	control (%)	3μM (%)	6μM (%)	12μM (%)	16μM (%)
Healing rate (%)	23.95401	14.27353	18.88755	16.27149	8.345724
	36.60708	22.46095	20.01065	19.14226	10.54731
	44.88873	24.29293	21.20226	19.69687	12.51959

Table S27 transwell migration rate

neferine	control (%)	3μM (%)	6μM (%)	12μM (%)	16μM (%)
migration rate (%)	100	92.90909	77.81818	56	7.272727
	100	61.37761	45.01845	28.04428	9.348093
	100	63.82979	38.82979	21.2766	9.574468

Table S28 transwell invasion rate

neferine	Control (%)	3μM (%)	6μM (%)	12μM (%)	16μM (%)
invasion rate (%)	100	75.41371	58.47458	23.87707	20.45455
	100	75.61983	53.90071	27.47934	21.75141
	100	87.57062	67.56198	21.75141	22.93144

Figure S3 p-MYPT1

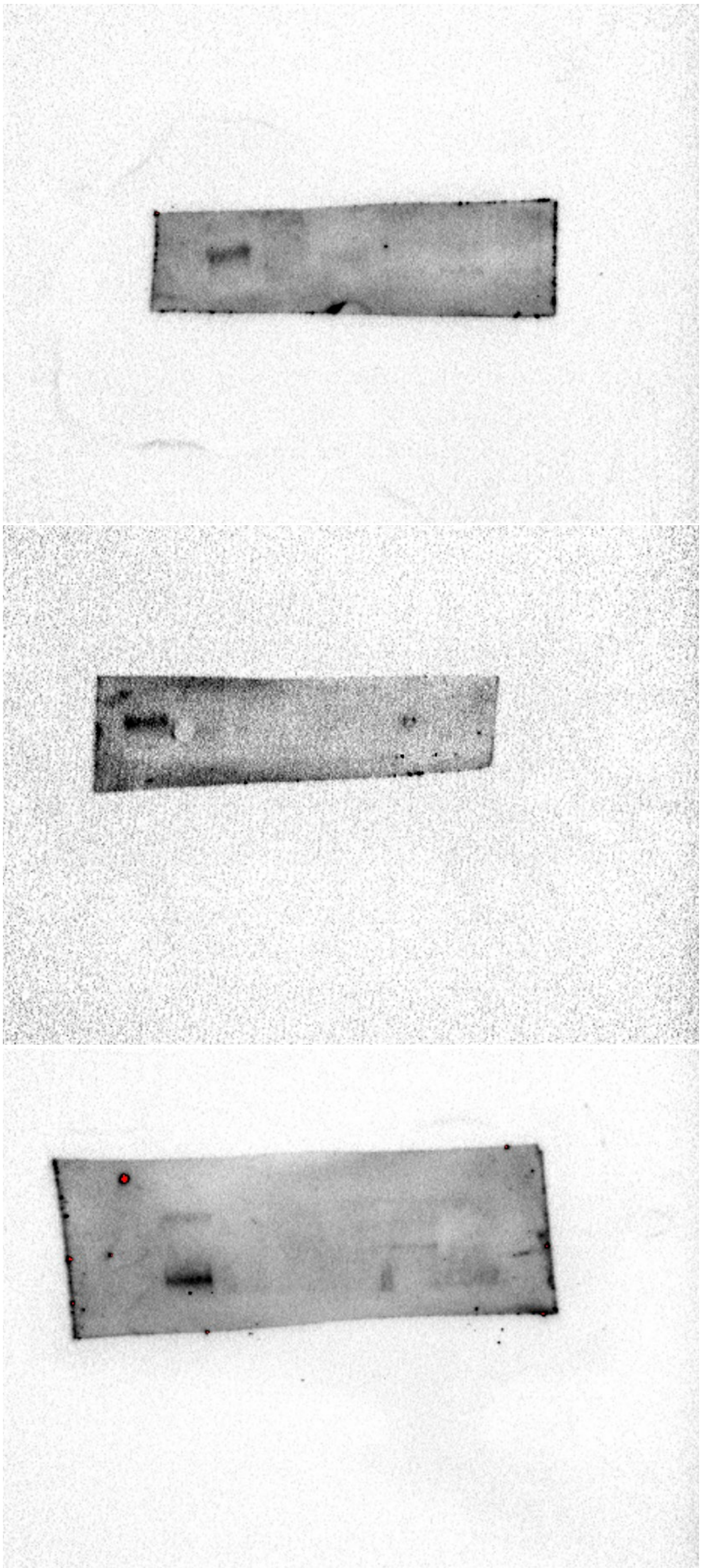

Figure S4 RhoA

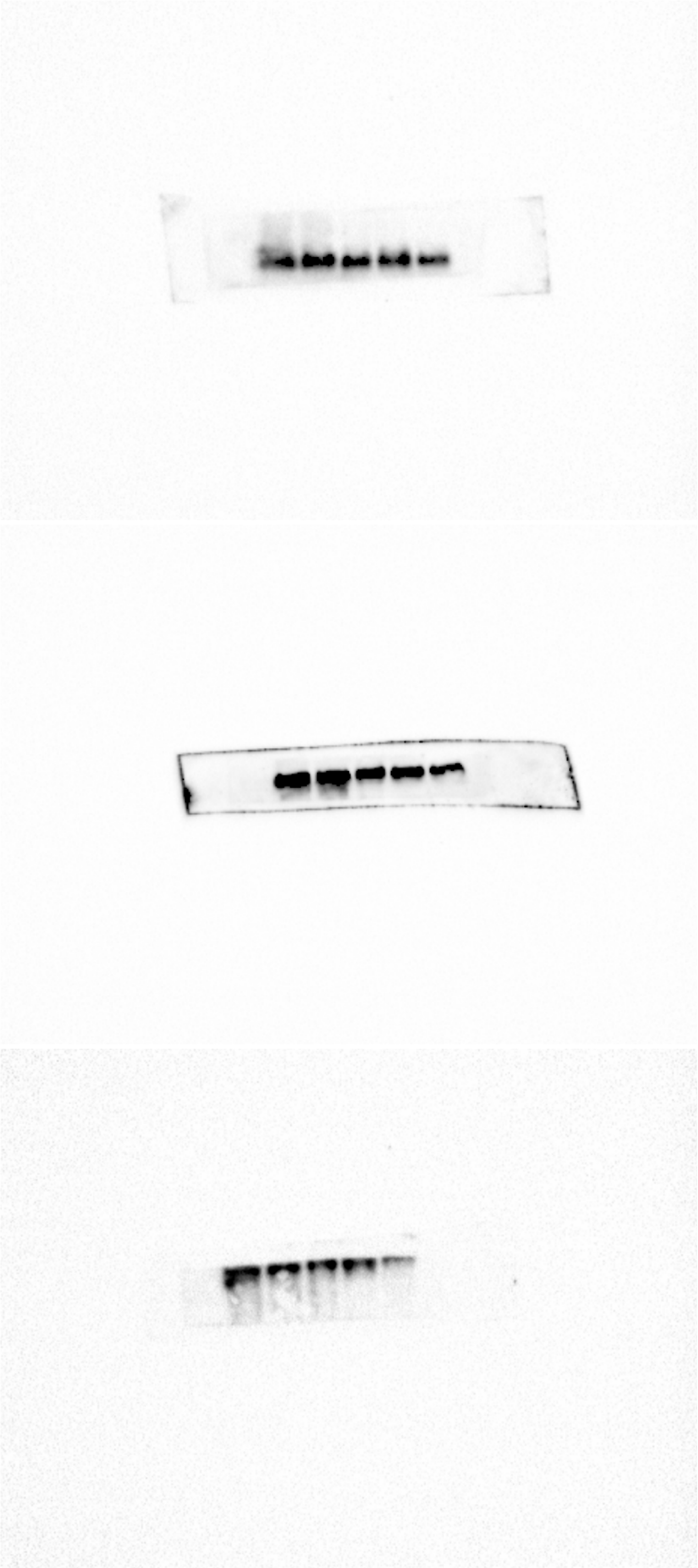

Figure S5 Vimentin

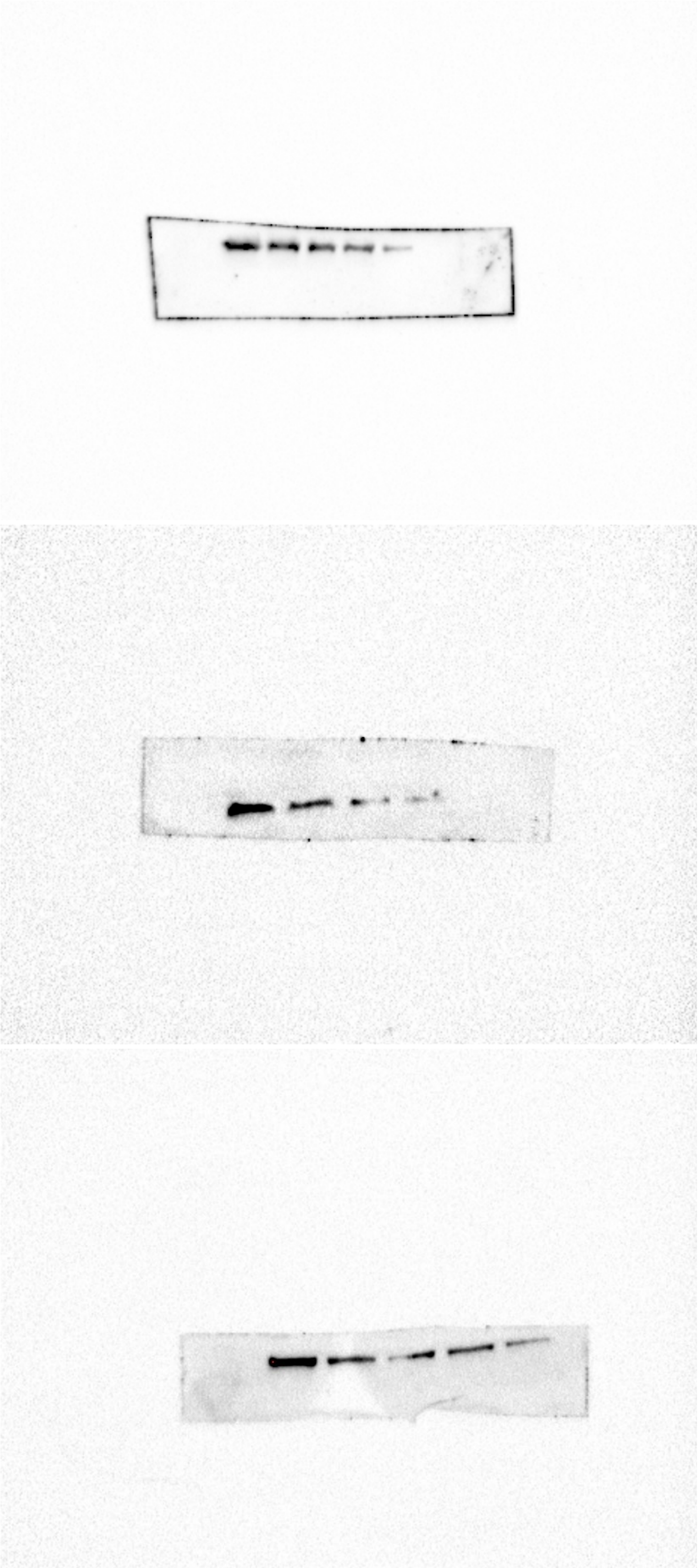

Figure S6 β-Actin

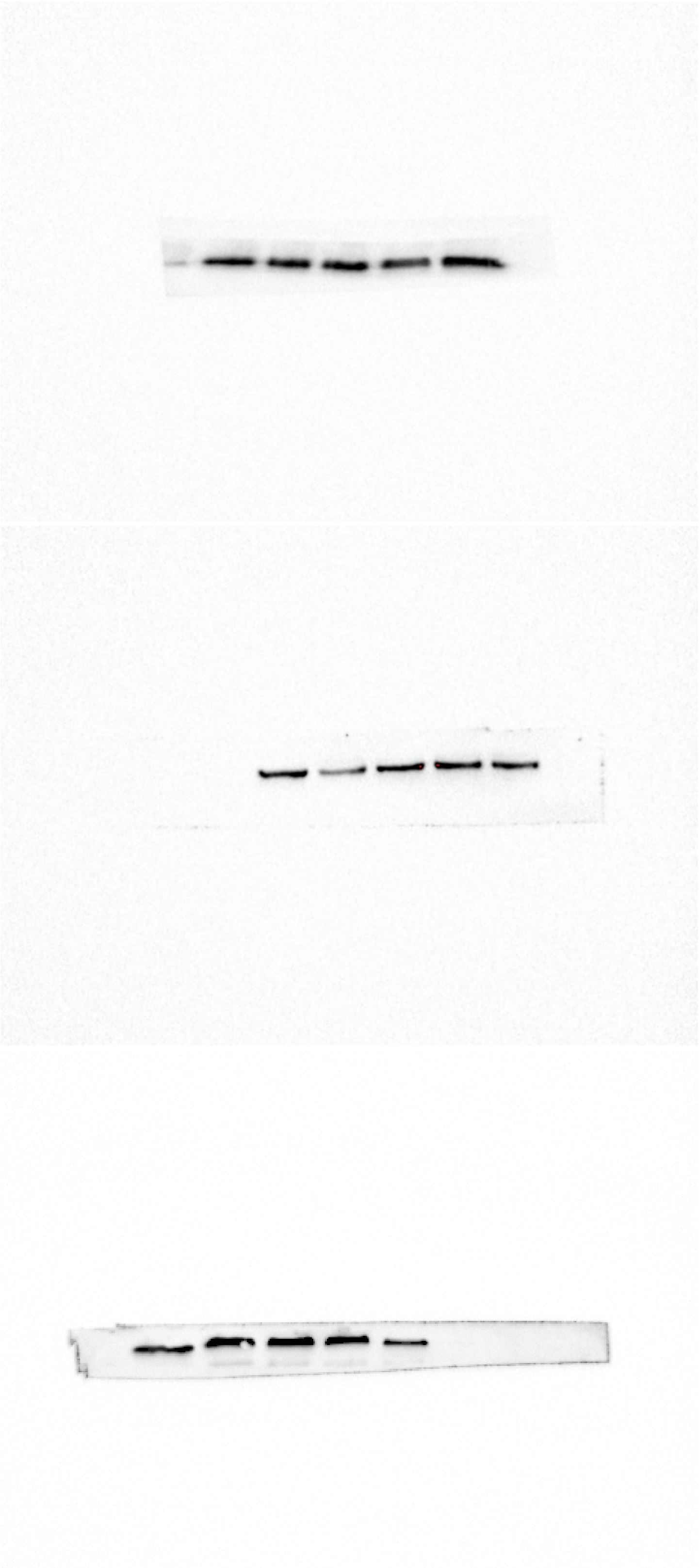

Table S29 Grayscale analysis of RhoA

0 μM	3 μM	6 μM	12 μM	16 μM
100	85.39866	75.92388	80.58412	54.47836
100	71.23806	64.11899	63.13025	24.90397
100	80.37463	46.59624	71.16335	34.92277

Table S30 Grayscale analysis of Vimentin

0 μM	3 μM	6 μM	12 μM	16 μM
100	70.01	51.88	38.31	14.11
100	49.83	40.91	47.22	28.02
100	50.38	23.26	17	8.23

Table S31 Hydrogen spectrum data of neferine

No.	δH (500 MHz; MeOD)	δH (Ref)[21]
1	3.65 (1H, m)	3.62 (1H, m)
1'	3.70 (1H, m)	3.62 (1H, m)
2-NCH3	2.48 (3H, s)	2.48 (3H, s)
2'-NCH3	2.52 (3H, s)	2.45 (3H, s)
3	2.74 (1H, m)<br>3.14 (1H, m)	2.73 (1H, m)<br>3.13 (1H, m)
3'	2.86 (1H, m)<br>3.03 (1H, m)	2.73 (1H, m)<br>3.08 (1H, m)
4	2.63 (1H, m)<br>2.77 (1H, m)	2.62 (1H, m)<br>2.79 (1H, m)
4'	2.54 (1H, m)<br>3.12 (1H, m)	2.59 (1H, m)<br>2.78 (1H, m)
5	6.65 (1H, s)	6.61 (1H, s)
5'	6.25 (1H, s)	6.49 (1H, s)
6-OCH3	3.76 (3H, s)	3.79 (3H, s)
6'-OCH3	3.74 (3H, s)	3.76 (3H, s)
7'-OCH3	3.46 (3H, s)	3.52 (3H, s)
8	6.90 (1H, s)	6.34 (1H, s)
8'	6.16 (1H, s)	5.97 (1H, s)
9	2.88 (1H, m)<br>2.85 (1H, m)	2.77 (1H, m)<br>2.98 (1H, m)
9'	2.68 (1H, m)<br>3.09 (1H, m)	2.67 (1H, m)<br>3.06 (1H, m)
11	6.86 (1H, s)	6.89 (1H, s)
11'	6.57 (1H, s)	6.51 (1H, s)
12	6.67 (1H, d)	6.67 (1H, d, J = 7.06 Hz)
13-OCH3	3.68 (3H, s)	3.7 (3H, s)
14	6.78 (1H, d)	6.67 (1H, d, J = 7.06 Hz)
14'	6.84 (1H, d)	6.82 (1H, d, J = 6.68 Hz)
15	6.66 (1H, d)	6.89 (1H, d, J = 7.06 Hz)
15'	6.80 (1H, d)	6.66 (1H, d, J = 6.68 Hz)
